# Role of the JAK-STAT Pathway in Bovine Mastitis and Milk Production

**DOI:** 10.3390/ani10112107

**Published:** 2020-11-13

**Authors:** Muhammad Zahoor Khan, Adnan Khan, Jianxin Xiao, Yulin Ma, Jiaying Ma, Jian Gao, Zhijun Cao

**Affiliations:** 1 State Key Laboratory of Animal Nutrition, Beijing Engineering Technology Research Center of Raw Milk Quality and Safety Control, College of Animal Science and Technology, China Agricultural University, Beijing 100193, China; zahoorkhattak91@163.com (M.Z.K.); dairyxiao@gmail.com (J.X.); ma18810318038@163.com (Y.M.); majiaying@cau.edu.cn (J.M.); 2 Key Laboratory of Animal Genetics, Breeding, and Reproduction, Ministry of Agriculture & National Engineering Laboratory for Animal Breeding, College of Animal Science and Technology, China Agricultural University, Beijing 100193, China; dr.adnan93@cau.edu.cn; 3 Department of Clinical Veterinary Medicine, College of Veterinary Medicine, China Agricultural University, Beijing 100193, China; gaojian2016@cau.edu.cn

**Keywords:** bovine mastitis, JAK-STAT pathway, *JAK2*, *STATs*, *SOCS3*, immunity, milk production

## Abstract

**Simple Summary:**

The cytokine-activated Janus kinase (JAK)—signal transducer and activator of transcription (STAT) pathway has an important role in the regulation of immunity and inflammation. In addition, the signaling of this pathway has been reported to be associated with mammary gland development and milk production. Because of such important functions, the JAK-STAT pathway has been widely targeted in both human and animal diseases as a therapeutic agent. Recently, the *JAK2*, *STATs*, and inhibitors of the JAK-STAT pathway, especially cytokine signaling suppressors (SOCSs), have been reported to be associated with milk production and mastitis-resistance phenotypic traits in dairy cattle. Thus, in the current review, we attempt to overview the development of the JAK-STAT pathway role in bovine mastitis and milk production.

**Abstract:**

The cytokine-activated Janus kinase (JAK)—signal transducer and activator of transcription (STAT) pathway is a sequence of communications between proteins in a cell, and it is associated with various processes such as cell division, apoptosis, mammary gland development, lactation, anti-inflammation, and immunity. The pathway is involved in transferring information from receptors on the cell surface to the cell nucleus, resulting in the regulation of genes through transcription. The Janus kinase 2 (*JAK2*), signal transducer and activator of transcription A and B (STAT5 A & B), STAT1, and cytokine signaling suppressor 3 (*SOCS3*) are the key members of the JAK-STAT pathway. Interestingly, prolactin (Prl) also uses the JAK-STAT pathway to regulate milk production traits in dairy cattle. The activation of *JAK2* and *STATs* genes has a critical role in milk production and mastitis resistance. The upregulation of *SOCS3* in bovine mammary epithelial cells inhibits the activation of *JAK2* and *STATs* genes, which promotes mastitis development and reduces the lactational performance of dairy cattle. In the current review, we highlight the recent development in the knowledge of JAK-STAT, which will enhance our ability to devise therapeutic strategies for bovine mastitis control. Furthermore, the review also explores the role of the JAK-STAT pathway in the regulation of milk production in dairy cattle.

## 1. Introduction

Bovine mastitis is a seriously infectious and contagious disease, which is a massive threat to the dairy industry throughout the globe [1]. Mastitis is the inflammation of the mammary gland, which is characterized by physical, chemical, and microbiological alterations in milk, following pathological changes in udder tissue [2]. Bovine mastitis is described as acute or chronic based on inflammation, redness, and localized heat at the infected area, with more severe symptoms, such as fever, leading to septicemia, and the formation of abscesses [3,4]. There are two types of mastitis: clinical and subclinical mastitis. In most cases, infection with Gram-negative bacteria such as *Escherichia coli* (*E. coli*) can often cause clinical mastitis, and Gram-positive bacteria such as *Staphylococcus aureus* (*S. aureus*) are involved in subclinical mastitis infection [5,6]. 

Bovine mastitis is considered one of the costly diseases of dairy cattle because of milk losses, treatment costs, and rare death [7,8]. In China, the annual losses of 15–45 billion Chinese Yuan (CNY) have been documented [7], while in the US and India, the dairy industry has experienced losses of 2 billion and 526 million dollars, respectively [9]. In Europe, collectively, the cost due to mastitis has reached 1.55 billion euros per year [10]. This increased frequency was linked to public concerns for animal welfare and has made mastitis the key disease of the dairy sector [11]. In addition, bovine mastitis has a major zoonotic risk, correlated with the shedding of bacteria and their toxins into milk [12].

Mammary epithelial cells are the first line of defense of the mammary gland to invading bacteria. They not only act as physical barriers but also are capable of producing inflammatory mediators. While interacting with invading bacteria, mammary epithelial cells generate multiple inflammatory cytokines [13,14]. Several genes and pathways have been reported to be associated with the regulation of bovine mastitis [15]. It is well known that the innate immune system recognizes the presence of pathogens ligands through a membrane receptor family known as Toll-like receptors (TLRs) [16]. TLRs are pattern recognition receptors (PRRs) on the host cell surface that recognize bacterial-pathogen-associated molecular patterns [17]. Upon activation, TLRs further mediate different important signaling, such as that of the JAK-STAT pathway. 

Any disruption in the JAK-STAT pathway may lead to various diseases, including bovine mastitis that compromises the immune system of the host. Furthermore, it has also been documented that *STAT5A* works as a mediator for extracellular prolactin receptors. At the same time, *JAK2* plays a role as a bridge between *STAT5A* and prolactin receptor (PrlR), which is essential for milk production and mammary gland development. Keeping in view the vital role of JAK-STAT signaling in immunity, inflammation, and milk production, the current review paper is designed with aims to summarize the role of the JAK-STAT pathway in bovine mastitis and milk production.

## 2. General Mechanism of the JAK-STAT Pathway Regulation

**Figure 1:**
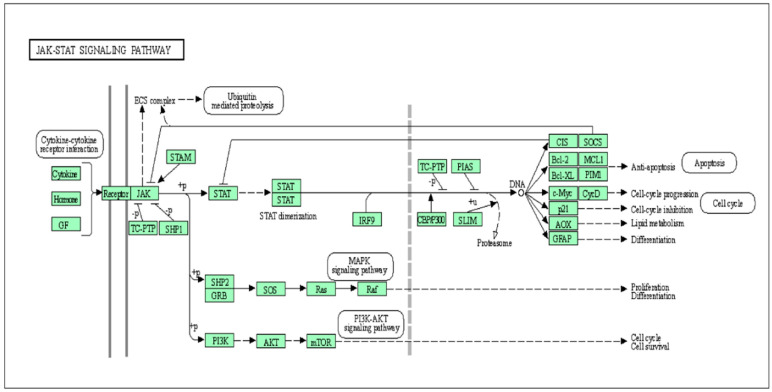
The regulation of the cytokine-activated Janus kinase (JAK)–signal transducer and activator of transcription (STAT) pathway by cytokines, hormones, and growth factors; engagement of the JAK-STAT pathway in the process of differentiation, survival, and proliferation through the modification in cellular gene expression.

There are three main components of the JAK-STAT pathway: receptors, Janus kinases (JAKs), signal transducers, and activators of transcription proteins (*STATs*) [18]. The mammalian JAK family consists of *JAK1*, *JAK2*, *JAK3*, and tyrosine kinase 2 (*TYK2*), which are linked to the cytoplasmic domains of diverse cytokine receptors [19]. Among the seven members of *STATs* (*STAT1*-4, 5a, 5b, and 6) in mammalian cells, *STAT5A* and *STAT5B* show high sequence identity and lie closest in a head-to-head pattern next to *STAT3* [19,20]. The members of the STAT family are involved in cell growth, differentiation, cell survival and apoptosis, and mammary gland development. The cytokines, after attachment with receptors on the cell surface, activate JAKs. The two JAKs come close through receptor oligomerization. Furthermore, these JAKs phosphorylate the receptor complex’s intracellular tyrosines, generating the docking sites for STATs. Consequently, the activated *STATs* form hetero- or homodimers, where the Src-homology 2 (SH2) domain of each STAT binds the phosphorylated tyrosine of the opposite STAT, and the dimers then translocate to the cell nucleus to induce transcription of the target genes. JAK-STAT has been revealed to operate downstream of several peptide hormones and cytokines that are necessary for the development of the postnatal and secretory function of the mammary gland [21]. The phosphorylated *STAT5A* and *STAT5B* form homodimers and heterodimers in mammary epithelial cells in order to regulate the process of differentiation, survival, and proliferation through the modification in cellular gene expression [22]. The rapamycin target phosphatidylinositol 3-kinase-protein kinase B/mammalian signaling pathway (PI3K-Akt/mTOR) mediates many cellular processes such as cell proliferation, growth, survival, and metastasis [23], and it is necessary for the development of the mammary gland [24]. A conditional knockout of Akt1 prevents the extensive survival of mammary epithelial cells, which express hyperactive *STAT5,* indicating that the PI3K-Akt/mTOR pathway is a crucial downstream signaling effector of JAK-STAT signaling [25]. To find out the interconnection between different genes and their biological functions in the JAK-STAT pathway, we exploited an online software database for annotation, visualization, and integrated discovery (DAVID; https://david.ncifcrf.gov/) [26], which are summarized in Figure 1. 

*STAT5*, being the main gene of the JAK-STAT inflammatory signaling pathway, has an essential role in prolactin-induced mammary gland factor and is assumed to be associated with mammary gland development in transgenic mice [27]. Consequently, upon activation, JAK regulates the cellular mechanisms such as cell migration, apoptosis, cell proliferation, and differentiation, which are essential for hematopoietic responses, immune development, mammary gland development, and the lactation process [28]. Cytokines play a vital role in the regulation of the JAK-STAT pathway, which further facilitates immunity and inflammation. Consequently, the JAK-STAT pathway has been widely studied for its critical role in immunity and inflammation [29,30], and evidence indicates that persistent activation of this pathway might lead to many immune- and inflammatory-related diseases [31,32]. Performing a critical role in immunity, cell proliferation, cell differentiation, and inflammation, the JAK-STAT pathway has been widely targeted for therapeutic purposes in several inflammatory diseases [33].

## 3. The JAK-STAT Pathway Role in Milk Production in Dairy Cattle

The JAK-STAT pathway regulates lactation [34], while PI3K/Akt within the JAK-STAT pathway shows overexpression in lactating cows [35]. Gene deletion analysis in mice has documented an important role of the JAK-STAT signaling pathway in the lactation and development of the mammary gland [36,37]. In the mammary gland, the JAK-STAT pathway, along with *SOCS* signaling, plays a critical role in controlling cytokine signals and has shown an association with mammary gland development and milk production [38]. Moreover, studies have documented the essential role of the JAK-STAT pathway in blood cell differentiation and casein gene regulation during milk production [39,40]. It has been shown that some JAK-STAT-associated proteins are regulated by PrlR, which may establish a balance between growth hormone and milk protein yield [41]. It has been illustrated that by using the JAK-STAT pathway, the lactogenic hormones, through their receptors on cell membranes, regulate milk proteins [42]. Prolactin also uses JAK-STAT signaling and regulates the processes of lactation and reproduction in mammals [43]. It has been documented that a higher concentration of Prl in blood circulation is associated with an increased level of milk production in dairy cattle [44]. During hypothyroidism, a severe decrease in milk production has been documented. Furthermore, it has been found that hypothyroidism decreases the level of prolactin, resulting in lower expression of the JAK-STAT pathway, which is responsible for lower milk production in hypothyroidized rats [45].

### 3.1. Role of JAKs in Milk Production in Dairy Cattle

JAK2 is the tyrosine kinase responsible for phosphorylation of both PrlR and Stat5, based on tissue culture cell studies. According to one report, in the absence of the *JAK2* gene, mammary epithelium proliferation and differentiation were reduced by 95% around parturition [46]. The endocrine factor prolactin attaches to the PrlRs and causes their dimerization. JAK protein kinases are linked to these receptors and these JAK proteins alter a receptor into a tyrosine kinase receptor. The regulated receptors may specifically phosphorylate inactive *STATs*, which result in dimerization. These dimers are further translocated into the nucleus. The *STATs* attach to the upstream promoter elements of the casein gene and cause their transcription. Growth hormones (GHs) control the growth and development of the mammary gland and regulate milk production and milk protein levels in cattle [41,47]. *STAT5* passes on messages from cytokines and growth factors outside the cell to the nucleus of the mammary gland epithelial cells and thereby mediates the transcription of the gene during pregnancy, lactation, and weaning [48]. 

It has been consistently reported that the polymorphisms T-C39652459 and T-C39645396, at intron 15 and exon 20, respectively, in the *JAK2* gene, are significantly associated with milk lactose production in dairy cows [49]. Furthermore, the variant *JAK2*/*RsaI* is involved in the regulation of milk and milk protein and can be considered a milk production marker in dairy cattle [50]. The variants 39630048C/T and 39631175T/C in the *JAK2* gene significantly influence milk fat and milk proteins, respectively, in Chinese Holsteins [51]. PrlR uses *STAT5A* and *JAK2* as mediators to activate the proteins associated with milk production traits [52].

### 3.2. Role of STATs in Milk Production in Dairy Cattle

STATs are activated by specific ligands, i.e., *STAT5A* is regulated by Prl, while STAT5B regulation is mediated through growth hormones (GHs) [53]. STAT5 is an important intracellular mediator of prolactin signaling and can activate transcription of milk proteins in response to Prl. *STAT5* has been suggested to be candidate marker genes for milk protein yield and composition in dairy cattle [54]. During pregnancy, *STAT5A* and PrlR play essential roles in mammary epithelium proliferation and differentiation [55,56]. Consequently, it has been found that PrlR has a positive impact on lactation performance in cows, possibly due to its involvement in steroid synthesis and cholesterol regulation [57]. During pregnancy and lactation, STAT5A and STAT5B are the essential proteins required for the synthesis of luminal progenitor cells from mammary stem cells and the differentiation of milk-producing alveolar cells [58]. *STAT5A* and *STAT5B* have been linked with the development of the mammary gland during pregnancy [59]. It was previously found that *STATs* promote the mammary gland cells’ survival by mediating the promoters of genes essential for milk proteins [34,60]. *STATs* facilitate various peptide hormones and cytokines in targeted cells such as Prl and GH and are linked to milk production. Whey acidic protein (WAP) is expressed in the mammary gland and is associated with the improvement of milk protein. *STAT5* has been considered an important transcription factor that is responsible for the regulations of Prl at 5′ flanking regions of *WAP* [61]. It has been observed that the downregulation of Prl in hypothyroidized rats causes the inhibition of the transcriptional activity of *STAT5.* Consequently, any abnormality in the thyroid gland severely affects milk production efficiency in rats because of the low level of Prl [45]. In addition, GH also regulates the STAT1 gene and its expression has been reported during mammary gland development [62,63]. Furthermore, a study has reported the combination effect of STAT1 with other JAK-STAT signaling members on milk production traits [38]. Keeping in view the important role of *STATs* as a mediator of prolactin signaling, the polymorphisms in these genes were further studied for its role in milk production. 

The mutations in the *STAT5A* gene have been reported for their effect on milk yield [64]. Consistently, the *STAT5A/AvaI* polymorphism at position C-T 6853/exon7 was documented to be associated with milk production and could be used as a significant marker for milk improvement [65]. In addition, the *STAT5A/MslI* locus has been found to be correlated with milk yield, milk fat, and protein [65,66,67]. The polymorphic site A14217G and 17266indelCCT in *STAT5A* have shown significant associations with milk protein percentage and milk yield, respectively [68]. Consequently, Schennink et al. documented that single nucleotide polymorphism (SNP) 9501G>A in *STAT5A* significantly influenced milk fat composition [69]. Khatib et al. noticed that variant 12195T/C in *STAT5A* was significantly linked to a decrease in milk fat and protein percentage in dairy cattle [70]. The variant 31562 T>C in *STAT5B* was reported to be associated with milk yield and milk protein [71]. The association of *CD4* and *STAT5B* with milk traits might be due to their role in the regulation of prolactin-induced mammary gland factor [72]. Moreover, the variant in the STAT 1 gene has been documented to be linked with milk fat, milk protein, and milk yield in dairy cattle [73]. Consequently, the polymorphism STAT1/BspHI has been reported to be associated with milk production traits in Jersey cows [74]. Similarly, Deng et al. reported that SNPs in STAT1 have a significant association with milk production traits and could be a useful addition to the marker-assisted selection for milk production [75].

The above findings reveal that the JAK-STAT pathway plays a central role in the regulation of milk production traits.

## 4. The JAK-STAT Signaling Role in Bovine Mastitis

As mastitis is an immunity- and inflammatory-related disease, scientists have widely targeted the JAK-STAT pathway in bovine mastitis control research. Besides having a critical role in mammary gland development, any abnormal regulation may disturb the normal function of the JAK-STAT pathway, resulting in impairment of mammary gland development and exposure to mammary infections. Buitenhuis et al. found the altered expression of the JAK-STAT pathway in the mammary gland tissue of cows challenged with *E. coli* [76]. It is well known that the JAK-STAT pathway is regulated by IFN, LPS, or growth factors. In its turn, JAK-STAT signaling mediates proinflammatory cytokines. Tiezzi et al. documented the JAK-STAT pathway as a key pathway that regulates clinical mastitis [77]. Recently, it has been reported that cirsimarin (an extract of *Cirsium japonicum* var. ussuriense) treatment suppressed the expression of inflammatory cytokines by downregulating the phosphorylation of the JAK-STAT pathway in the mammary gland. Thus, this substance can be targeted as a therapeutic agent in many inflammatory diseases, including bovine mastitis [78]. It has been shown that 8-methoxypsoralen treatment protects bovine mammary epithelial cells against lipopolysaccharide-induced inflammatory injury by inhibition of the JAK/STAT and NF-κB pathways [79]. JAK-STAT suppression by xanthotoxin resulted in the downregulation of IL-6, nitric oxide (NO), and tumor necrosis factor (TNF-α) induced by LPS in bovine mammary epithelial cells [80]. This mechanism is essential for regulating udder reactions to infection as it controls the chronic accumulation of neutrophils in the bovine mammary gland [81], whereas JAK also serves as a signaling agent for hormones and interleukin receptors [82] and *JAK2* is considered one of the top-rated genes of bovine mastitis tolerance [83]. 

### 4.1. Association of JAK2 Activity with Bovine Mastitis

Any dysfunctions of the JAK-STAT pathway may expose cattle to mastitis because of abnormal activation of the proliferation and apoptosis of cells. From this point of view, it can be expected that mutations in genes involved in the JAK-STAT pathway might be a target in bovine mastitis research. The inflammatory- and immunity-associated diseases are polygenic traits [71], and polymorphisms in immunity-linked genes can regulate the immune responses of the host to pathogens [84]. Two major approaches are dominantly targeted by animal scientists to control mastitis. The first approach is to look for major genes associated with mastitis resistance, while the second one is to target the polymorphisms within genes and their links with mastitis resistance traits.

Many types of mutations in the JAK-STAT pathway have been identified; most of them are related to *JAK2*. 

As demonstrated in Table 1, the polymorphism 39630048C/T in *JAK2* is associated with interleukin-17 (IL-17) [85], IL-6, and interferon-gamma (IFN-γ) expression [51]. Furthermore, the SNPs (39652267A/G, 39631175T/C) in the *JAK2* gene have been documented for their significant links with milk somatic cell counts (SCCs), IL-6, and IFN-γ [51,85]. Mutation 39631044G/A in the *JAK2* gene was noticed to be significantly associated with milk somatic cell scores (SCSs) in Chinese Holsteins [85]. Moreover, the polymorphism 39645396C/T in the *JAK2* gene was linked to milk SCCs, IL-6, and IFN-γ [86], while SNP-39631044G/A in *JAK2* was associated with milk SCSs [85]. SCCs and SCSs are widely targeted as early mastitis indicators [7]. Increased SCCs in early lactation can signify the presence of intramammary infection, and, in many countries, the indirect selection against mastitis using milk SCCs is practiced [87]. However, in the early phase of infectivity, the neutrophil and inflammatory cytokine levels increase quicker than milk SCCs [88]. That is why, nowadays, more interest is paid to the increase in cells and cytokine levels in milk and blood, respectively, rather than just the overall SCC, which may provide an early status of udder health [89]. A study showed that inflammatory cytokines (IL-6, IL-17, and IFN-γ, TNF-α) could be used as subclinical mastitis indicators, in addition to SCSs and SCCs [51,86,90]. In addition, it is predicted that the 39645396C/T SNP changes lysine to asparagine [86]. The expression of IL-6 was higher in plasma cell mastitis (PCM), which indicated that the IL-6/STAT3 pathway could play a key role in the pathogenesis of PCM [22,91]. The IL-17 family consists of cytokines that participate in acute and chronic inflammation and provoke the host’s defense against microbial organisms [92]. T-helper 17 cells are thought to be a significant source of IL-17A; furthermore, IL-17, producing innate immune cells, activate the fast release of IL-17A [93] in response to pathogens or tissue injury [94]. 

IL-17 has been shown to be significantly upregulated in goat milk infected with *E. coli* or *S. aureus* [95]. *IL-17A* production was documented during *S. uberis* mastitis [96], and slightly increased expression was also noticed in the somatic cells of cows infected with *S. aureus* [97]. Furthermore, an in-vitro study illustrated that IL-17A reinforces the ability of mammary epithelial cells (MECs) to resist the consequences produced by *S. aureus* [98]. It has been reported that *IL-17A* and *IL-17F* play a critical role in regulating host–pathogen interactions during the development of mastitis [99]. The SNPs in *IL-17F* and *IL-17A* have been shown to be associated with milk SCCs [90]. Moreover, IL-17 also activates IL-6 with IFN-γ and tumor necrosis factor-alpha (TNF-α) [100]. Usman et al. revealed that IL-6 is the best indicator of mastitis and can be a target in mastitis control strategies [85]. Altogether, the above-published studies show that IL-17, IL-6, IL-4, IFN-γ, SCS, and SCC are the key indicators of mastitis. The interactions of polymorphisms in *JAK2* with bovine mastitis resistance phenotypic traits (IL-17, IL-6, IL-4, IFN-γ, SCS, and SCC) show that *JAK2* might be considered a useful marker in bovine mastitis resistance strategies. 

### 4.2. Role of STATs in Bovine Mastitis

A variety of cytokines and growth factors activate *STATs*, which are a family of latent transcription factors. During the process of inflammation, *STAT5B* regulates CD4+ T-cells differentiation [101]. STAT1 raised the expression of *SOCS3* and SOCS1 in *S. aureus*-infected mammary epithelial cells [102]. Furthermore, it was reported that upon treatment with JAK inhibitors, the plasma cells in PCM decreased considerably due to the suppression of IL6/STAT/JAK signaling, resulting in the reversion of pathogenesis [91]. Accordingly, it was found that the inflammatory cytokines regulate the JAK-STAT pathway in the mammary gland; in response, the phosphorylation of STAT takes place. The phosphorylated STAT translocates into the nucleus and mediates the production of proinflammatory genes that facilitate mastitis’s pathogenesis [78]. It is known that the inflammatory cells are recruited towards the site of infection, in which T-cells, particularly CD4+ cells, are predominantly observed in bovine mastitis [103]. Rivas et al. revealed that *S. aureus*-infected dairy cows showed a remarkable elevation in the level of CD4+ T-cells at the early stage of infection in the mammary gland [104]. Eder’s team recently proved that the CD4+T-cell level was higher in dry cows compared to lactating cows. These findings show that a decrease in the level of CD4+ T-cells in lactating dairy might be one of the reasons for susceptibility to infection during this stage [105]. Usman et al. reported a significant association of variant T104010752C in the *CD4* gene with milk SCCs [90]. In the previous study, it was noticed that polymorphisms in *CD4* and *STAT5B* genes are significantly linked with mastitis-resistance phenotypic traits [83]. Furthermore, the polymorphism in *CD4* at locus g.13598C>T showed a significant association with SCS, which is the crucial indicator of mastitis. 

The combination geneotype analysis of CD4 g.13598C>T and STAT5b g.31562 T>C is associated with milk SCSs in Chinese Holsteins. Furthermore, it was reported that cows with combination genotypes of CCTT show the highest estimated breeding value (EBV) for SCSs [71]. Another study documented that the silencing of the *CD4* gene through DNA methylation influences the progress of CD4+ T-cells in inflammatory conditions [106]. These findings demonstrate that CD4 protein and CD4+ T-cells play essential roles in host defense during the development of mastitis.

As demonstrated in Table 2, the polymorphism in *STAT5A* (43046497A/C) is associated with IL-6 and also changes the amino acid isoleucine to valine [85]. Similarly, mutation at point 43673888A>G in the *STAT5B* gene was significantly linked to mastitis-resistance phenotypic traits (IL-4 and SCC) [86]. Bochniarz et al. reported the elevated level of IL-6 and decreased level of IL-4 in the milk and serum of cows infected with *S. aureus* [107]. In addition, the polymorphism STAT5A-AvaI was associated with milk SCCs and electrical conductivity (EC) in the milk of mastitic cows [108]. EC in milk is one of the essential indicators of bovine mastitis because of its association with Na and Cl levels, which increase during mastitis. Cai et al. also reported a STAT5A gene through genomewide association studies (GWAS) as a potential candidate marker for bovine mastitis resistance [109]. Based on the above-published findings, we concluded that *STAT5A* and *STAT5B* might be target mastitis-resistance markers in dairy cattle. 

## 5. Inhibitors of the JAK-STAT Pathway: Role in Mastitis and Milk Production

The protein inhibitors of activated STAT (PIAS) [110], protein tyrosine phosphatases (PTPs) [111], and cytokine signaling suppressors (SOCSs) [112] are three major classes used by cells to control the JAK-STAT pathway [113]. PIAS proteins are considered important transcriptional coregulators of JAK-STAT signaling because of their significant contribution to the control of gene expression [114]. PIAS proteins restrict the regulation of the JAK-STAT pathway in three ways: (1) by adding a small ubiquitin-like modifier (SUMO) group to STAT and blocking its phosphorylation, (2) by preventing the binding of STAT to DNA [115], and (3) by recruiting histone deacetylase to remove acetyl changes to histones by lowering gene expression [116]. Similarly, PIAS3, a member of the PIAS family, has been identified to inhibit STAT3 signaling after regulation by the cytokine IL-6 [117]. Moreover, PIAS1 could inhibit NF-κB and JAK-STAT activity regulated by cytokine TNF and the LPS endotoxin [110]. PIAS has a major role in cell proliferation [118], cell apoptosis, and the immune response [115]. Protein tyrosine phosphatases (PTPs) are a group of enzymes that remove the phosphate group from the JAK-STAT pathway and prevent the action of signaling [119]. The *STATs* are deactivated by PTPS in both the nucleus and cytoplasm. Src homology phosphatase 2 (SHP-2) is one of the members of PTPs that inactivate STAT5 in the cytoplasm. Similarly, SHP1 prevents the phosphorylation of the JAK-STAT pathway and blocks its further action [120,121]. The general role of JAK-STAT inhibitors has been summarized by recently published reviews in more detail [31,122]. Although the two groups of PTPs and PIAS have essential roles in the regulation of the JAK-STAT pathway, their tasks have not been evaluated in milk production or bovine mastitis to date. Therefore, we have only focused on cytokine signaling suppressors (SOCSs) in our current review. 

Some SOCS proteins are triggered by cytokines and pathogenic mediators and, thus, function in a classical negative-feedback loop to impede the transduction of cytokine signals. Consequently, they represent an effective mechanism for the negative regulation of the cytokine-mediated JAK-STAT pathway [123]. The DNA binding of STAT protein regulates the mRNA expression of SOCSs [124]. *SOCS3* can inhibit JAK tyrosine kinase activity directly via its kinase-inhibitory region (KIR), which has been proposed to serve as a pseudosubstrate and is essential for cytokine signal suppression [125]. Undeniably, both a KIR and a KIR-mimetic peptide, classified as the tyrosine kinase inhibitor peptide (TKIP), have been described to inhibit *JAK2*-regulated transcription factor STAT1 phosphorylation [126,127]. The SH2 domain of SOCS can also directly bind to the receptors and prevent the signal from passing to JAK-STAT signaling [128]. Moreover, Kimura et al. revealed that LPS could activate *JAK2* and *STAT5*, which participate in the induction of IL-6, while SOCS1 inhibits this process selectively [129].

The suppression of IL-6 and IFN-γ usually occurs around parturition, which depresses immunity and exposes dairy cattle to mastitis [130]. Normal levels of IL-6 and IFN-γ are necessary for the maintenance of bovine immunity. Moreover, *SOCS3* has been reported to be one of the key inhibitors of IL-6 and IFN-gamma. This evidence shows that *SOCS3* might have a potential role in mastitis development in dairy cattle [131]. Moreover, Fang et al. found that *SOCS3* was significantly upregulated after the mammary gland had been infected with *S. aureus*. The authors further supposed that *SOCS3* could negatively regulate the JAK-STAT pathway, which might be one of the reasons for its critical role in mastitis development [132]. Huang et al. also reported that *SOCS3* is a negative regulator of the JAK-STAT pathway. Furthermore, it was demonstrated that overexpression and inhibition of *SOCS3* brought visible changes in milk protein, which might be due to the action of *SOCS3* on the JAK-STAT pathway [133]. The Huang team further suggested that a low level of *SOCS3* is essential for the regulation of milk synthesis. Similarly, a study reported that *SOCS3* inhibits the induction of Prl and activation of STAT5 [134]. Zahoor et al. found that merTK reduces the inflammatory changes induced by *S. aureus* through *STATs*/SOCS3 signaling [102]. Furthermore, it has been revealed that impaired SOCS1/3 has a crucial role in the susceptibility of mammary epithelial cells to *S. aureus* infections. Additionally, a study reported a polymorphism in SOCS2, which was significantly associated with susceptibility to inflammation of the mammary gland [135]. *SOCS3* also has an inhibitory role in STAT5 regulation, which is one of the strong reasons for their influence on lactational performance in dairy cattle. Further study is highly recommended to find out the specific variants in *SOCS3* that interact with *STAT5* and *JAK2* during mastitis development and milk production in dairy cattle. 

## 6. Conclusions

Altogether, it can be concluded that a delicate equilibrium must be achieved for the effective activation of the JAK/STAT pathway, when the immune system is needed for action against infection, and proper restoration when the infection is diminished. Thus, the JAK-STAT pathway can be considered as a therapeutic option in mastitis control and enhancement of milk production strategies. Furthermore, it is suggested that the interactive mechanism of *SOCS3*, *STATs*, and *JAK2*, *STAT5A*, and *STAT5B* during milk production and mastitis development should be considered in future rodent-knockout research models. It is highly recommended that further polymorphisms in STAT1 and *SOCS3* and their associations with milk production and mastitis resistance traits be found out. Finally, PTPs and PIAS are critical inhibitors of the JAK-STAT pathway, so research on the evaluation of their role in bovine mastitis would be an interesting development.

## Figures and Tables

**Table 1:** Association of SNPs in *JAK2* with bovine mastitis resistance phenotypic traits.

Gene	Mutation	Reference	Position	Phenotypic Traits	Authors
*JAK2*	C-T/EXON16	rs210148032	Chr8:39652267	SCC	[51,85]
*JAK2*	C-T/EXON20	rs110298451	Chr8:39645396	IL-6, IFN-γ, SCC	[51,86]
*JAK2*	C-T/3′flanking region	rs135128681	Chr8:39630048	IL-6, IFN-γ, SCC	[51,85]
*JAK2*	T-C/3′flanking region	Novel	Chr8:39631175	IL-6, SCC	[51,85]
*JAK2*	G-A/3′flanking region	Novel	Chr8:39631044	SCS	[85]
*JAK2*	5′ upstream	rs379754157	Chr8:39750638	SCC	[49]

**Table 2:** Association of SNPs in *STAT5A* and *STAT5B* with bovine mastitis resistance phenotypic traits.

Gene	Mutation	Reference	Position	Phenotypic Traits	Authors
*STAT5A*	A-C/Intron 9	rs109358395	Chr19:43046497	IL-6	[85]
*STAT5B*	A-G/Intron 4	rs41915686	Chr19:43673888	IL-4, SCC	[86]
*STAT5b*	T-C/EXON 8		Chr19:31562	SCS	[71]
